# Enhanced drought and salt stress tolerance in Arabidopsis via ectopic expression of the *PvMLP19* gene

**DOI:** 10.1007/s00299-025-03520-y

**Published:** 2025-05-22

**Authors:** Bayram Ali Yerlikaya, Seher Yerlikaya, Abdullah Aydin, Nisa Nur Yilmaz, Sibel Bahadır, Mohamed Farah Abdulla, Karam Mostafa, Musa Kavas

**Affiliations:** 1 Department of Agricultural Biotechnology, Faculty of Agriculture, Ondokuz Mayis University, Samsun, 55270 Turkey; 2 East Precision Agriculture and Innovation Technology LLC, Abu Dhabi, UAE; 3 The Central Laboratory for Date Palm Research and Development, Agricultural Research Center (ARC), Giza, 12619 Egypt

**Keywords:** Climate resilience, Common bean, Drought tolerance, PvMLP19, Root development, Seed germination

## Abstract

**Key message:**

*PvMLP19* overexpression in Arabidopsis enhances proline accumulation, mitigates oxidative stress, improves water retention, delays germination, and stimulates root growth under drought and salt stress conditions.

**Abstract:**

Climate change has exacerbated the frequency and severity of drought and salinity stress, posing significant risks to agricultural productivity and food security. As sessile organisms, plants have evolved regulatory mechanisms to adapt to these challenges. Common bean (*Phaseolus vulgaris* L.), an essential legume crop valued for its high nutritional value, is increasingly impacted by climate change-induced stressors. The PR10 protein family has been recognized as a potential contributor to enhancing plant resilience to abiotic and biotic stresses. This family, also known as Bet v1, is highly conserved and consists of diverse subfamilies, including major latex proteins (MLPs), which may contribute to stress tolerance through ligand-binding and regulation of stress-related pathways. This study aimed to investigate the functional role of *PvMLP19* in stress tolerance using both in silico and experimental approaches. RNA-seq analysis revealed tissue-specific expression patterns of PR10s, with PvMLP19 showing notable induction under abiotic stress. Functional validation in transgenic Arabidopsis suggested that overexpression of *PvMLP19* may improve drought tolerance. Transgenic plants exhibited increased proline accumulation, reduced oxidative stress, and higher relative water content under both drought and salinity stress conditions. Furthermore, *PvMLP19* overexpression was associated with delayed seed germination but promoted root development under osmotic and salinity stress. The increased stress tolerance was linked to the upregulation of stress-inducible genes, suggesting a potential regulatory role of PvMLP19 in modulating stress-response pathways. These findings position PvMLP19 as a potential candidate for genetic improvement in crops, offering a promising strategy to mitigate the impacts of climate change and ensure sustainable agricultural productivity.

**Supplementary Information:**

The online version contains supplementary material available at 10.1007/s00299-025-03520-y.

## Introduction

Global climate models forecast a notable rise in the frequency and intensity of hot and dry days, posing a significant threat to crop yield and quality (Lawas et al. 2018; Yerlikaya et al. 2020). As water scarcity intensifies and the global population continues to grow, drought has become a critical challenge to agricultural productivity (Hu and Xiong 2014; Mostafa et al. 2024). Due to their sessile nature, plants have developed intricate regulatory mechanisms to adapt and respond to environmental stresses effectively.

Common beans (*Phaseolus vulgaris* L.) are highly valued as a staple grain legume. They are renowned for their exceptional protein content and wealth of essential micronutrients, making them a critical component of human diets (Broughton et al. 2003). However, their cultivation is increasingly threatened by the detrimental impacts of climate change, which disrupt agricultural productivity. To mitigate these challenges, common beans have evolved complex adaptive and responsive mechanisms that enable them to endure and adapt to a variety of environmental stresses. (Lone et al. 2021). One potential component of these adaptive mechanisms is the PR10 protein family, which plays a vital role in enhancing tolerance to both biotic and abiotic stresses. Due to their multifunctional nature, these proteins are recognized as one of the key contributors to multi-stress resilience in plants (Agarwal and Agarwal 2014). The PR10 protein family, also known as the Bet v 1 family, is categorized into 11 distinct subfamilies based on their functional roles and similarities in amino acid sequences. These subfamilies include classic PR10 proteins, major latex proteins (MLP), cytokinin receptors, and plant polyketide cyclase-like proteins (Radauer et al. 2008).

The Bet v1 protein family is highly conserved across various plant species and plays critical roles in stress tolerance and plant development. In walnut, for example, Bet v1-like PR10 proteins have been found to interact with chitinases, thereby strengthening defense responses against anthracnose, highlighting their role in biotic stress resistance (Wang et al. 2025). A newly identified PR10/Bet v1-like subfamily member was also implicated in pathogen resistance in rice (Li et al. 2022). Moreover, studies have characterized related genes in other species, including the PgPR10-4 gene in *Panax ginseng*, the NCSI gene in sacred lotus (*Nelumbo nucifera*), and the VASt gene in *Arabidopsis thaliana*, each contributing to stress responses and physiological regulation (Kim et al. 2014; Khafif et al. 2014; Vimolmangkang et al. 2016). Together, these findings illustrate the widespread conservation and multifaceted roles of the Bet v1 protein family in enhancing plant adaptation to stress and supporting developmental processes. PR10/Bet v1 proteins primarily function as ligand-binding molecules, interacting with diverse compounds such as cytokinins, brassinolides, and secondary metabolites. These ligand–protein interactions are crucial for triggering downstream signal transduction pathways, which regulate crucial physiological processes and stress responses in plants (Koistinen et al. 2005; Radauer et al. 2008). Notably, in *A. thaliana*, the abscisic acid (ABA) receptor RCAR/PYR/PYL family of START domain proteins shares significant amino acid and structural similarities to Bet v1 proteins, underscoring the functional diversity and evolutionary relevance of this family (Park et al. 2009). PR10 proteins have been shown to play significant roles in responses to both abiotic and biotic stresses (Liu and Ekramoddoullah 2006; Yuan et al. 2020; Holmquist et al. 2021). For instance, overexpression of the *GhMLP28* gene from cotton in Arabidopsis improved salt stress tolerance (Chen and Dai 2010), while its expression in *Nicotiana tabacum* enhanced resistance to *Verticillium dahliae* infection (Yang et al. 2015). Similarly, overexpression of *AtMLP43* was reported to enhance drought tolerance through the ABA signaling pathway (Wang et al. 2016a, b). 

Members of the PR10 family have been identified in *Phaseolus vulgaris*, with a genome-wide analysis by Feki et al. (2024) identifying 34 *PR10* encoding genes grouped into three subgroups: 21 *PvMLP*, 2 *PvPBP*, 1 *PvNCS*, and 10 *PvPR10* members. This study highlighted the evolutionary diversity of PR10-related proteins in common bean, yet their precise biological functions remain unclear, necessitating further functional characterization to improve stress tolerance in crops. PvPR10 genes also exhibit diverse expression profiles across developmental stages, tissues, and organs. Several PvPR10 genes are significantly upregulated in leaves and roots under various abiotic stresses and phytohormonal treatments (Feki et al. 2024).

In this study, we conducted an in silico analysis of RNA-seq data to evaluate the response of PR10 genes to drought and salt stress in roots, leaves, and hypocotyls, focusing on their tissue-specific expression patterns. Based on this analysis, we identified and selected *PvPR10*-*1*, *PvNCS*, *PvMLP19*, and *PvMLP21* for qPCR validation in common bean roots subjected to abiotic stress treatments. Among these, the gene *Phavu_011G183900* (*PvMLP19*) was strongly induced under both salt and drought conditions. Subsequently, the full-length cDNA of *PvMLP19* was cloned, confirming that it encodes a novel PR10 protein. To gain further insights into the regulatory mechanisms driving *PvMLP19* expression under salt and drought stress, we employed transgenic Arabidopsis plants. The possible role of *PvMLP19* on seed germination and root formation was evaluated using transgenic Arabidopsis plants.

## Materials and methods

### Plant materials and applied stress conditions

The Ispir genotype, a salinity-tolerant common bean (*Phaseolus vulgaris* L.) variety widely cultivated in Türkiye, was selected for abiotic stress experiments in this study. Seeds were surface sterilized with a 5% (v/v) sodium hypochlorite solution for 10 min, rinsed three times with sterile distilled water, and sown in 1-L pots filled with autoclaved vermiculite. Following germination, plants were cultivated in a controlled growth chamber at 24 °C, 60% relative humidity, and a 16-h light/8-h dark photoperiod with a light intensity of 250 µmol m⁻^2^ s⁻^1^ provided by cool white fluorescent lamps. Plants were irrigated twice weekly with 50 mL of half-strength Hoagland’s solution (pH 6.0) under optimal conditions. Stress treatments were applied to 4-week-old plants as follows: drought stress was induced by supplementing the irrigation solution with 20% (w/v) polyethylene glycol (PEG) 6000 (average molecular weight 6000 Da), and salinity stress was imposed using 200 mM sodium chloride (NaCl) in the same solution. For hormone-induced stress, leaves were sprayed with 100 μM abscisic acid (ABA) or 100 μM indole-3-butyric acid (IAA), each dissolved in distilled water with 0.02% (v/v) Tween-20 to enhance adhesion. Control plants were maintained under optimal irrigation with half-strength Hoagland’s solution without additives. Root samples from stressed, hormone-treated, and control plants were harvested at 6-, 12-, 24-, and 48-h post-treatment, immediately frozen in liquid nitrogen, and stored at − 80 °C for subsequent RNA extraction.

### Expression analysis in common bean

Raw RNA-seq data for analyzing PvPR10 gene expression across various tissues and stress conditions were retrieved from the Sequence Read Archive (SRA). The dataset comprised eight distinct comparisons: (1) leaf explants of salt-sensitive (T43) and salt-tolerant (Ispir) common bean genotypes under salt stress (PRJNA656794); (2) root samples from the same genotypes under identical salt stress conditions (PRJNA656794); (3) root tissues of the salt-tolerant Ispir genotype under control (irrigated with Hoagland’s solution) versus 200 mM NaCl stress (PRJNA656794); (4) bud-stage tissues of salt-tolerant and salt-sensitive genotypes under salt stress (PRJNA558376); (5) sprout-stage tissues of the salt-tolerant Ispir genotype under 200 mM NaCl stress versus control (PRJNA691982); (6) drought-tolerant Pinto Saltillo genotype under control versus drought stress (PRJNA508605); (7) drought-tolerant Perola genotype under control versus drought stress (PRJNA327176); and (8) common bean plants infected with *Sclerotinia sclerotiorum* (strain 1980) versus uninfected controls (PRJNA574280). Tissue-specific expression profiles of *PvPR10* genes were sourced from Phytozome v12.1 (*Phaseolus vulgaris* genome v2.1). Transcriptome analysis was conducted using Galaxy (usegalaxy.eu) platforms. Reads were aligned to the *P. vulgaris* reference genome (v2.1) using HISAT2 (v2.1.0) with default parameters (–dta option enabled for downstream transcript assembly). Transcript assembly was performed with StringTie (v1.3.3) using a minimum isoform abundance of 0.1, followed by differential expression analysis with DeSEq2. Genes with a log_2_ fold change (FC) > 1 and a *p*-value < 0.05 (adjusted for multiple testing via the Benjamini–Hochberg method) were classified as differentially expressed. A heatmap of log_2_ FC values was generated using the pheatmap package in R (v4.2.2) with hierarchical clustering based on Euclidean distance.

RNA was extracted from root tissues of Ispir plants subjected to stress (200 mM NaCl, 20% PEG 6000) and hormone treatments (100 μM ABA, 100 μM IAA) using the RNeasy Plant Mini Kit (Qiagen, Hilden, Germany) according to the manufacturer’s instructions. RNA quality, integrity, and concentration were evaluated using a NanoDrop 2000/2000c spectrophotometer (Thermo Fisher Scientific) and 1% (w/v) agarose gel electrophoresis stained with ethidium bromide. First-strand cDNA was synthesized from 1 μg of total RNA using the iScript cDNA Synthesis Kit (Bio-Rad, Hercules, CA, USA).

**Fig. 1:**
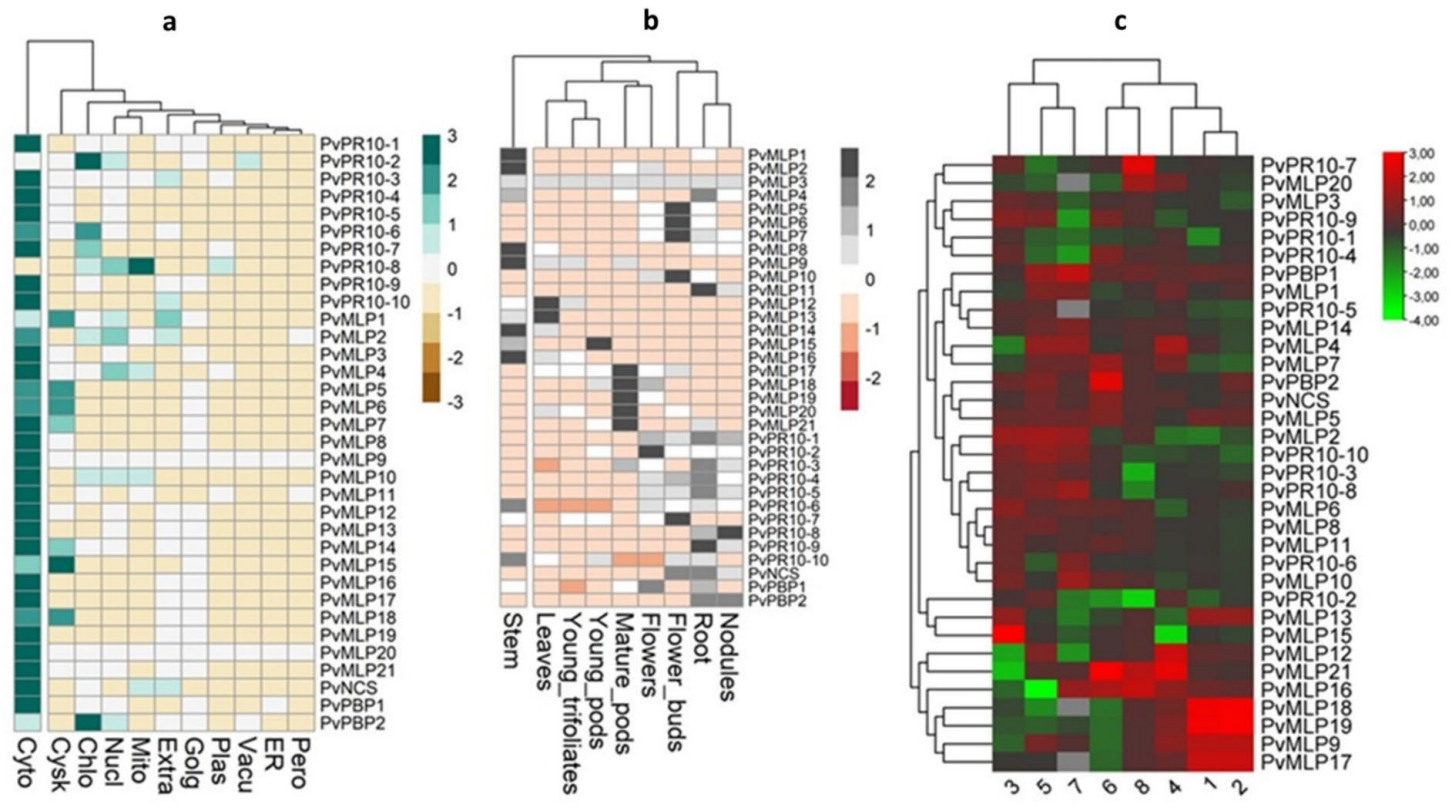
Subcellular, tissue-specific, and stress-responsive expression profiling of the PvPR10 family in common bean. The predicted subcellular localization of 34 PvPR10 proteins was determined using the TargetP-2.0 server and visualized as a heatmap generated with the R package pheatmap (v1.0.12). Rows represent individual PvPR10 genes, and columns denote subcellular compartments (e.g., cytosol, mitochondria, chloroplasts, cytoskeleton). Color intensity indicates localization probability, with brighter shades (yellow) reflecting higher predicted abundance and darker shades (blue) indicating lower probability. (b) Tissue-specific expression patterns of 34 PvPR10 genes across six common bean organs (leaves, stems, roots, flower buds, pods, nodules), derived from Phytozome v12.1 RNA-seq data. Expression levels (log2-transformed FPKM) are visualized as a heatmap using pheatmap, with hierarchical clustering (Euclidean distance, complete linkage) delineating two major clusters. The color scale ranges from blue (low expression) to red (high expression). (c) Stress-responsive expression profiles of PvPR10 transcripts under biotic and abiotic stress conditions, based on eight publicly available RNA-seq datasets. Differential expression (log_2_ FC) is shown as a heatmap, with blue indicating downregulation and red indicating upregulation (*p* < 0.05, log_2_ FC > 1). Datasets include: (1) leaves of salt-tolerant (Ispir) vs. salt-sensitive (T43) cultivars under 200 mM NaCl (PRJNA656794); (2) roots of the same cultivars under salt stress (PRJNA656794); (3) roots of Ispir under salt stress vs. control (PRJNA656794); (4) bud-stage samples of tolerant vs. sensitive cultivars under salt stress (PRJNA558376); (5) hypocotyls of Ispir under 12-h salt stress vs. control (PRJNA691982); (6) Perola cultivar under drought stress (150 min post-treatment) vs. control (PRJNA327176); (7) Pinto Saltillo cultivar under drought stress vs. control (PRJNA508605); (8) resistant cultivar infected with *Sclerotinia sclerotiorum* vs. non-infected control (PRJNA574280) (color figure online)

Four *PvPR10* genes (*PvPR10-1, PvNCS, PvMLP19,* and *PvMLP21*) were selected for qRT-PCR validation based on their differential expression patterns identified in RNA-seq analysis and their predicted roles in stress-responsive protein–protein interaction (PPI) networks. RNA-seq data (Fig. 1c) revealed that these genes were significantly upregulated in root tissues of the salt-tolerant Ispir genotype under 200 mM NaCl and 20% PEG6000-induced drought stress (log2 FC > 1, *p* < 0.05). Additionally, in silico PPI analysis using the STRING database indicated potential interactions of these genes with proteins involved in abiotic stress signaling pathways, such as ABA and auxin-related networks, with *PvMLP19* showing homology to Arabidopsis *AtMLP43*, a known regulator of drought tolerance.

Gene expression was quantified using an Agilent Mx3000P qRT-PCR system (Agilent Technologies, Santa Clara, CA, USA) with Solis BioDyne 5× HOT FIREPol EvaGreen qPCR Mix Plus (Solis BioDyne, Tartu, Estonia). Each reaction (20 μL) contained 1 μL cDNA, 0.5 μM of each primer, and 4 μL qPCR mix, with three biological and three technical replicates per condition. The thermal cycling protocol consisted of an initial denaturation at 95 °C for 2 min, followed by 40 cycles of 95 °C for 15 ss and 60 °C for 1 min, with fluorescence detection at the annealing/extension step. Relative expression levels were calculated using the 2^−∆∆*CT*^ method, normalized to the housekeeping gene *PvACT11* (Actin11). Primers for *PvPR10* genes and *PvACT11* (Table S1) were designed using NCBI Primer-BLAST (https://www.ncbi.nlm.nih.gov/tools/primer-blast/) to ensure specificity (amplicon size: 80–130 bp, *T*_m_: 58–62 °C). The comparative *C*_t_ method (Heid et al. 1996) was applied to determine relative expression ratios.

### Cloning of the *PvMLP19* gene and transformation of Arabidopsis through the floral dip method

The full-length coding sequence (CDS) of *PvMLP19* was amplified via reverse transcription polymerase chain reaction (RT-PCR) using total RNA extracted from *Phaseolus vulgaris* (Ispir genotype) roots as a template. RNA was isolated as described above, and first-strand cDNA was synthesized using the iScript cDNA Synthesis Kit (Bio-Rad, Hercules, CA, USA) with 1 μg of total RNA. Amplification was performed using gene-specific primers PvMLP19-Fw and PvMLP19-Rv (Table S1), designed to flank the CDS, in a 25 μL reaction containing 1 μL cDNA, 0.5 μM of each primer, 200 μM dNTPs, and 1 U Taq polymerase (Thermo Fisher Scientific). The PCR conditions were: initial denaturation at 95 °C for 3 min, followed by 35 cycles of 95 °C for 30 s, 58 °C for 30 s, and 72 °C for 1 min, with a final extension at 72 °C for 5 min. The resulting amplicon was purified using a QIAquick PCR Purification Kit (Qiagen, Hilden, Germany) and cloned into the pENTR/D-TOPO entry vector (Invitrogen, Carlsbad, CA, USA) following the manufacturer’s protocol. Successful cloning was verified by Sanger sequencing. For expression in plants, the PvMLP19 insert was transferred from the entry vector to the binary destination vector pIPKb004 using Gateway LR Clonase II Enzyme Mix (Invitrogen) via recombination at the attR1 and attR2 sites, placing PvMLP19 under the control of the constitutive CaMV 35S promoter. The recombinant pIPKb004-PvMLP19 plasmid was introduced into *Agrobacterium tumefaciens* (strain GV3101) by electroporation using a MicroPulser (Bio-Rad) set at 2.5 kV, 200 Ω, and 25 μF. Transformed Agrobacterium cells were selected on LB agar with 50 μg mL^−1^ kanamycin and confirmed by colony PCR using PvMLP19-specific primers. *Arabidopsis thaliana* (ecotype Col-0) plants were transformed using the floral dip method (Clough and Bent 1998). Briefly, flowering plants were dipped in an *Agrobacterium* suspension (OD600 = 0.8) containing 5% (w/v) sucrose and 0.02% (v/v) Silwet L-77 for 10 s, then incubated in the dark for 24 h before returning to standard growth conditions (22 °C, 16-h light/8-h dark). Transgenic seeds were harvested and screened on 1/2 MS medium supplemented with 50 μg mL^−1^ hygromycin to select T1 plants, which were advanced to the T3 generation for homozygosity.

### Root length and seed germination assays in PvMLP19 transgenic lines

To investigate the physiological role of PvMLP19 under abiotic stress, seed germination, and root length assays were conducted using wild-type (WT) Arabidopsis (Col-0) and three independent T3 transgenic lines overexpressing *PvMLP19* (#5, #8, #12). For germination assays, seeds were surface sterilized with 70% (v/v) ethanol for 1 min, followed by 5% (v/v) sodium hypochlorite for 5 min, and rinsed five times with sterile distilled water. Sterilized seeds (*n* = 16 per replicate, three biological replicates) were sown on solid 1/2 Murashige and Skoog (MS) medium (pH 5.8, 0.8% w/v agar) supplemented with mannitol (0, 100, 200, or 300 mM) or NaCl (0, 100, 150, or 200 mM) to simulate drought and salt stress, respectively. Plates were sealed, stratified at 4 °C in the dark for 2 days to synchronize germination, and then incubated at 25 °C in the dark. Germination, defined as radicle emergence, was recorded daily, with final rates and photographic documentation taken on day 7 post-sowing, following Ma et al. (2019) and Mostafa et al. (2024). For root length assays, WT and transgenic seeds were germinated on unsupplemented 1/2 MS medium for 3 days under standard conditions (22 °C, 16-h light/8-h dark, 250 μmol m⁻^2^ s⁻^1^). Uniform seedlings (*n* = 10 per replicate, three biological replicates) were transferred to 1/2 MS plates containing mannitol (0, 150, or 300 mM) or NaCl (0, 75, or 150 mM) and grown vertically for an additional 7 days. Primary root length was measured using ImageJ software (v1.53) by analyzing digital images of the plates, following protocols by Rushton et al. (2012), Seo et al. (2012), and Chen et al. (2015).

### Biochemical and expressional analysis of transgenic Arabidopsis plants under drought and salt stress

To evaluate drought stress tolerance of plants, seeds from wild-type (WT) *Arabidopsis thaliana* (Col-0) and T3-generation PvMLP19 overexpression transgenic lines (#5, #8, #12) were sown in a soil mixture (1:1 ratio of vermiculite and flower nutrient soil) in 200-mL pots. Plants were grown under controlled conditions (22 °C, 16-h light/8-h dark, 250 μmol m⁻^2^ s⁻^1^ light intensity, 60% relative humidity) and irrigated twice weekly with 20 mL of distilled water for 21 days to establish well-irrigated conditions. Drought stress was initiated on day 22 by withholding water for 4, 6, 8, or 10 days. On day 10 of the drought, plants were rewatered with 20 mL of distilled water daily for 4 days to assess recovery. Fresh weight was measured every 2 days during the drought period and post-rewatering using a precision balance, following protocols by Kang et al. (2019), Zhu et al. (2020), and Mostafa et al. (2024), to quantify drought tolerance. For salt stress, 21-day-old plants were irrigated with 20 mL of 200 mM NaCl solution every 2 days for 10 days, while controls received distilled water, adapting methods from Niu et al. (2012) and Ying et al. (2012). Post-treatment, aerial tissues (leaves and stems) were harvested, immediately frozen in liquid nitrogen, and stored at − 80 °C for RNA extraction. Total RNA was extracted using the RNeasy Plant Mini Kit (Qiagen, Hilden, Germany), and cDNA was synthesized from 1 μg RNA using the iScript cDNA Synthesis Kit (Bio-Rad, Hercules, CA, USA). Expression levels of drought- and salt-responsive genes (*ABI3, NCED, RD22, COR15 A*) were quantified via qRT-PCR on an Agilent Mx3000P system using primers listed in Table S1. Reactions (20 μL) contained 1 μL cDNA, 0.5 μM primers, and Solis BioDyne 5 × HOT FIREPol EvaGreen qPCR Mix Plus, with three biological and three technical replicates. The cycling protocol was 95 °C for 2 min, followed by 40 cycles of 95 °C for 15 s and 60 °C for 1 min. Expression was normalized to the housekeeping gene AtEF-1α (Czechowski et al. 2005) using the 2^−∆∆*CT*^ method, enabling a comparison of stress-induced changes between WT and transgenic lines. Free proline content, an osmotic stress indicator, was measured in leaf samples (100 mg fresh weight) using the ninhydrin-based method of Bates et al. (1973) and Kavas et al. (2016), with absorbance read at 520 nm. Malondialdehyde (MDA) content, a marker of lipid peroxidation, was quantified in 100 mg leaf samples via the thiobarbituric acid (TBA) method (Heath and Packer 1968; Kavas et al. 2016), with absorbance measured at 532 nm and corrected for nonspecific absorption at 600 nm. These assays provided insights into the physiological responses of PvMLP19 transgenic lines under abiotic stress.

### Exploring genetic networks: insights from co-expression analysis and ortholog discovery in Arabidopsis

To identify orthologs, bidirectional BLASTP alignments were performed between *Phaseolus vulgaris* (v2.1) and *Arabidopsis thaliana* (TAIR10) protein sequences using NCBI BLAST+ (v2.12.0) with an e-value cutoff of 1e-10. Orthologous pairs were determined by selecting the top reciprocal hit based on the highest BIT score. *PvMLP19* (Phvul.011G183900) was identified as orthologous to *AtMLP43* (AT1G70890), with a BIT score of 104 and an e-value of 9e-29. To explore functional relationships, a co-expression network for AtMLP43 was constructed using the ATTED-II NetworkDrawer tool (Obayashi et al. 2007; v11.0). Network parameters were set to ‘add a few genes’ (Pearson correlation coefficient > 0.7) for co-expression analysis and ‘Draw PPIs’ to visualize protein–protein interactions (PPIs) sourced from the STRING database (score > 400). The resulting network was exported as a Cytoscape-compatible file and annotated with log_2_ fold change values from qRT-PCR data.

### Statistical analysis

All experiments were conducted in three independent biological replicates, with data presented as means ± standard deviations (SD) depicted by error bars. Statistical analyses were performed using Microsoft Excel (v16.0). Differences between WT and transgenic lines were assessed with a two-tailed Student’s t test, with significance thresholds set at *p* < 0.05 (single asterisk, *) and *p* < 0.01 (double asterisks, **), as indicated in figure legends.

## Results

### Subcellular, tissue-specific, and stress-responsive expression profiling of *PvPR10* family genes in common bean

To characterize the *PvPR10* gene family, an in silico analysis was performed to investigate their subcellular localization, tissue-specific expression, and stress-responsive profiles, as depicted in Fig. 1. Subcellular localization of 34 PvPR10 proteins was predicted using the TargetP-2.0 server, with results visualized as a heatmap generated by the R package pheatmap (Fig. 1a). The heatmap illustrates relative protein abundance across compartments, with rows representing individual PvPR10 genes and columns indicating subcellular locations (e.g., cytosol, mitochondria, chloroplasts). Color intensity reflects localization probability, where brighter shades denote higher predicted concentrations. Most PvPR10 proteins were predominantly cytosolic, except PvPR10-8, localized to mitochondria, and PvPR10-2, associated with chloroplasts, while PvMLP1, PvMLP5, PvMLP6, PvMLP7, PvMLP14, PvMLP15, and PvMLP18 also showed cytoskeletal signals. Tissue-specific expression profiles of the 34 PvPR10 genes were analyzed using RNA-seq data from Phytozome v12.1, covering key common bean organs (leaves, stems, roots, flower buds, pods, nodules), and presented as a heatmap (Fig. 1b). Hierarchical clustering revealed two major groups: Cluster 1, with genes highly expressed in pods, leaves, and flowers (e.g., *PvMLP12* and *PvMLP13* in leaves), and Cluster 2, with genes preferentially expressed in flower buds, roots, and nodules (e.g., *PvMLP5* and *PvMLP7* in flower buds). Specific patterns included elevated expression of *PvMLP1*, *PvMLP2*, *PvMLP8, PvMLP9, PvMLP14,* and *PvMLP16* in stems, and *PvMLP1* and *PvMLP21* in pods. Stress-responsive expression was assessed using eight publicly available RNA-seq datasets (Fig. 1c), detailing responses to salt, drought, and biotic stress (*Sclerotinia sclerotiorum* infection). Notable findings included significant upregulation of *PvMLP9, PvMLP17, PvMLP18,* and *PvMLP19* in leaf and root tissues of salt-tolerant cultivars under 200 mM NaCl (log_2_ FC > 2, *p* < 0.05; PRJNA656794), with *PvMLP15* also induced in roots (log_2_ FC = 1.8, *p* < 0.05). *PvMLP16* showed upregulation under drought in the Perola cultivar (log_2_ FC = 2.3, *p* < 0.01; PRJNA327176), but downregulation in salt-stressed hypocotyls (log_2_ FC = − 1.5, *p* < 0.05; PRJNA691982). Conversely, *PvPR10-2* exhibited reduced expression under drought (log_2_ FC = − 1.7, *p* < 0.05) and increased expression in salt-stressed hypocotyls (log_2_ FC = 1.6, *p* < 0.05), indicating diverse regulatory responses across conditions and tissues.

### Analysis of PvPR10 transcript expression profiles in common bean under various abiotic and phytohormonal stress treatments

**Fig. 2:**
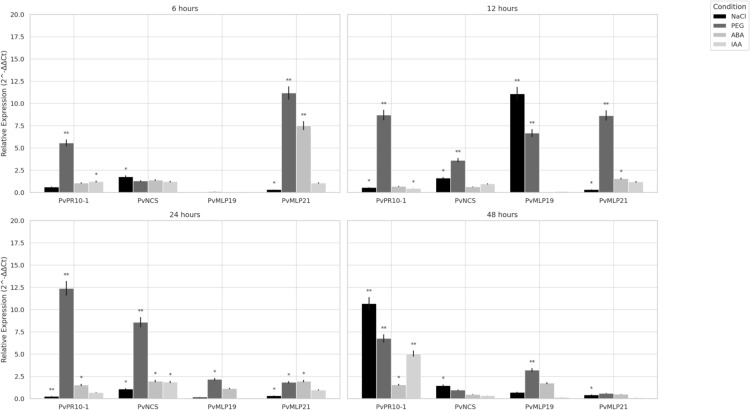
Expression analysis of *PvPR10* genes in common bean under diverse abiotic and phytohormonal stress conditions. The expression levels of four *PvPR10* genes (*PvPR10-1, PvNCS, PvMLP19,* and *PvMLP21*) were assessed through qRT-PCR following exposure to 200 mM NaCl, 20% PEG6000, 100 μM ABA, and 100 μM IAA. Samples treated with distilled water were used as controls. Root tissues were collected at four time intervals: 6-, 12-, 24-, and 48-h post-treatment. Error bars indicate standard deviations calculated from three independent biological replicates. Statistical significance was evaluated using Student’s *t* test, with *p < 0.05 and **p < 0.01 denoting significant and highly significant differences, respectively

To investigate the stress-responsive roles of *PvPR10* genes in *Phaseolus vulgaris* (Ispir genotype), transcript levels of four candidates, *PvPR10-1, PvNCS, PvMLP19,* and *PvMLP21*, were quantified via qRT-PCR under abiotic (200 mM NaCl, 20% PEG6000) and phytohormonal (100 μM ABA, 100 μM IAA) treatments, with expression assessed at 6-, 12-, 24-, and 48-h post-treatment (Fig. 2). Root tissues from treated and control plants (irrigated with distilled water) were analyzed with expression normalized to *PvACT11* using the 2^−∆∆CT^ method. *PvPR10-1* exhibited PEG-induced upregulation across all time points, with the highest expression at 24 h (12.38-fold), followed by 12 h (8.69-fold), 48 h (6.75-fold), and 6 h (5.56-fold). Its NaCl response was notably high at 48 h (10.67-fold), but low at earlier time points (e.g., 0.25-fold at 24 h). *PvNCS* showed PEG-induced expression at 12 h (3.61-fold) and 24 h (8.57-fold), but this response diminished by 48 h (0.95-fold). *PvMLP19* displayed a sharp NaCl-induced increase at 12 h (11.08-fold), with minimal expression at other time points (e.g., 0.01-fold at 6 h), while its PEG response was elevated at 12 h (6.66-fold) and 48 h (3.20-fold). *PvMLP21* exhibited strong early responses to PEG and ABA at 6 h (11.16-fold and 7.49-fold, respectively), with PEG expression remaining high at 12 h (8.63-fold), but decreasing at later time points (e.g., 0.57-fold at 48 h). NaCl consistently induced low expression in *PvMLP21* across all time points (0.32- to 0.41-fold). No consistent IAA-induced changes were observed across time points. These expression patterns suggest that *PvPR10-1* and *PvMLP21* might be involved in drought and ABA responses, particularly at early stages. *PvMLP19* could potentially contribute to salt and drought stress adaptation at specific time points. *PvNCS* may play a role in delayed drought responses, providing initial observations on the roles of *PvPR10* genes in abiotic stress contexts.

### Overexpression of *PvMLP19* modulates germination delay under mannitol and salinity stress conditions

**Fig. 3:**
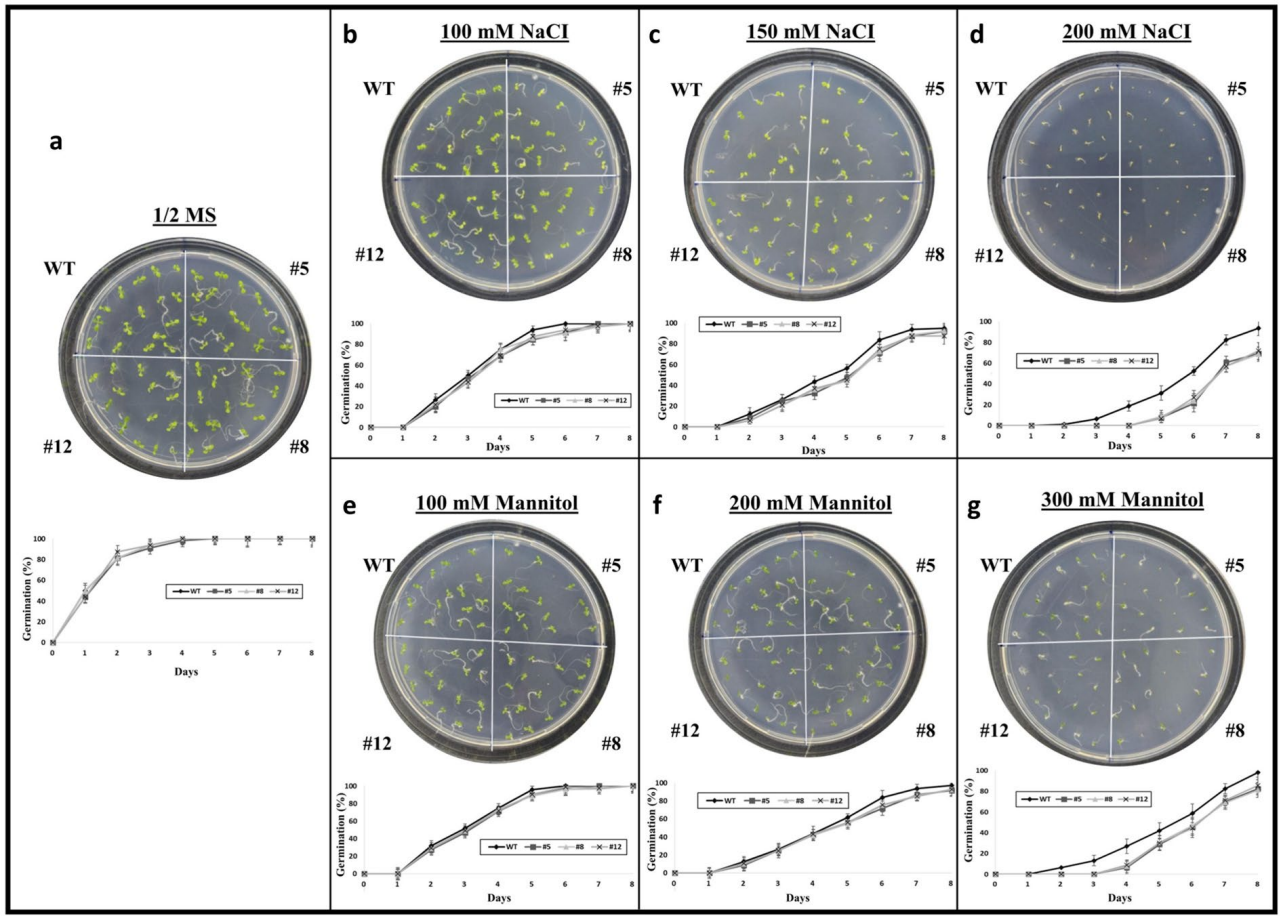
Overexpression of *PvMLP19* in Arabidopsis delays seed germination under osmotic stress conditions. **a** Germination under control conditions (1/2 MS). **b**–**d** Seed germination responses to salt treatments (100 mM, 150 mM, and 200 mM NaCl). **e**–**g** Germination under mannitol-induced osmotic stress (100 mM, 200 mM, and 300 mM mannitol). Petri dish images were captured at 48-h post-treatment to visually confirm germination differences. Seed germination performance was assessed for both WT plants and PvMLP19 transgenic lines (#5, #8, and #12) from the T3 generation, with quantitative rates recorded daily until day 7. Error bars indicate the mean ± standard deviation (SD) from three independent biological experiments (color figure online)

To investigate the physiological function of *PvMLP19*, we generated five independent transgenic Arabidopsis lines (#3, #5, #8, #11, and #12) that overexpress the PvMLP19 gene. The integration of the transgene was confirmed via PCR with *HPTII* gene-specific primers (Table S1, Fig. S1B). qRT-PCR-based expression analysis revealed several lines with substantially increased PvMLP19 transcript levels. Among these, lines #5, #8, and #12 from the T3 generation were selected based on their exceptional expression profiles (Fig. S1). These high-expression lines were used to evaluate the biological role of PvMLP19 in plant systems. To investigate the physiological function of *PvMLP19*, seed germination was assessed in WT and transgenic Arabidopsis lines (#5, #8, #12) under control and stress conditions (Fig. 3). Petri dish images, captured at 48-h post-treatment, visually confirmed germination differences, while quantitative germination rates were recorded daily until day 7. In 1/2 MS control medium, WT seeds exhibited 100% germination by day 4, while transgenic lines reached the same rate by day 5 (Fig. 3a). Under 100 mM NaCl, WT seeds achieved 100% germination on day 6, compared to day 7 for transgenic lines (Fig. 3b). At 150 mM NaCl, WT seeds reached full germination on day 7 and transgenic lines on day 8 (Fig. 3c). Under 200 mM NaCl stress, WT seeds exhibited a germination rate of 90% (approximately 14 out of 16 seeds, on average) by day 8, whereas transgenic seeds showed a reduced germination rate of 65% (around 10 out of 16 seeds, on average) (Fig. 3d). In response to mannitol-induced osmotic stress, WT seeds achieved complete germination by day 6 at 100 mM, day 7 at 200 mM, and day 8 at 300 mM concentrations (Figs. 3e, f, and g). Conversely, transgenic seeds reached full germination by day 8 only at 100 mM mannitol, while germination rates at 200 mM and 300 mM concentrations plateaued at 90% (approximately 14 out of 16 seeds, on average) and 75% (approximately 12 out of 16 seeds, on average), respectively (Fig. 3e, f, g). The consistent delay and reduction in germination across stress conditions suggest that *PvMLP19* overexpression may negatively impact germination under salinity and osmotic stress.

### Overexpression of *PvMLP19* enhances root development and lateral root formation under mannitol and salinity stress conditions

**Fig. 4:**
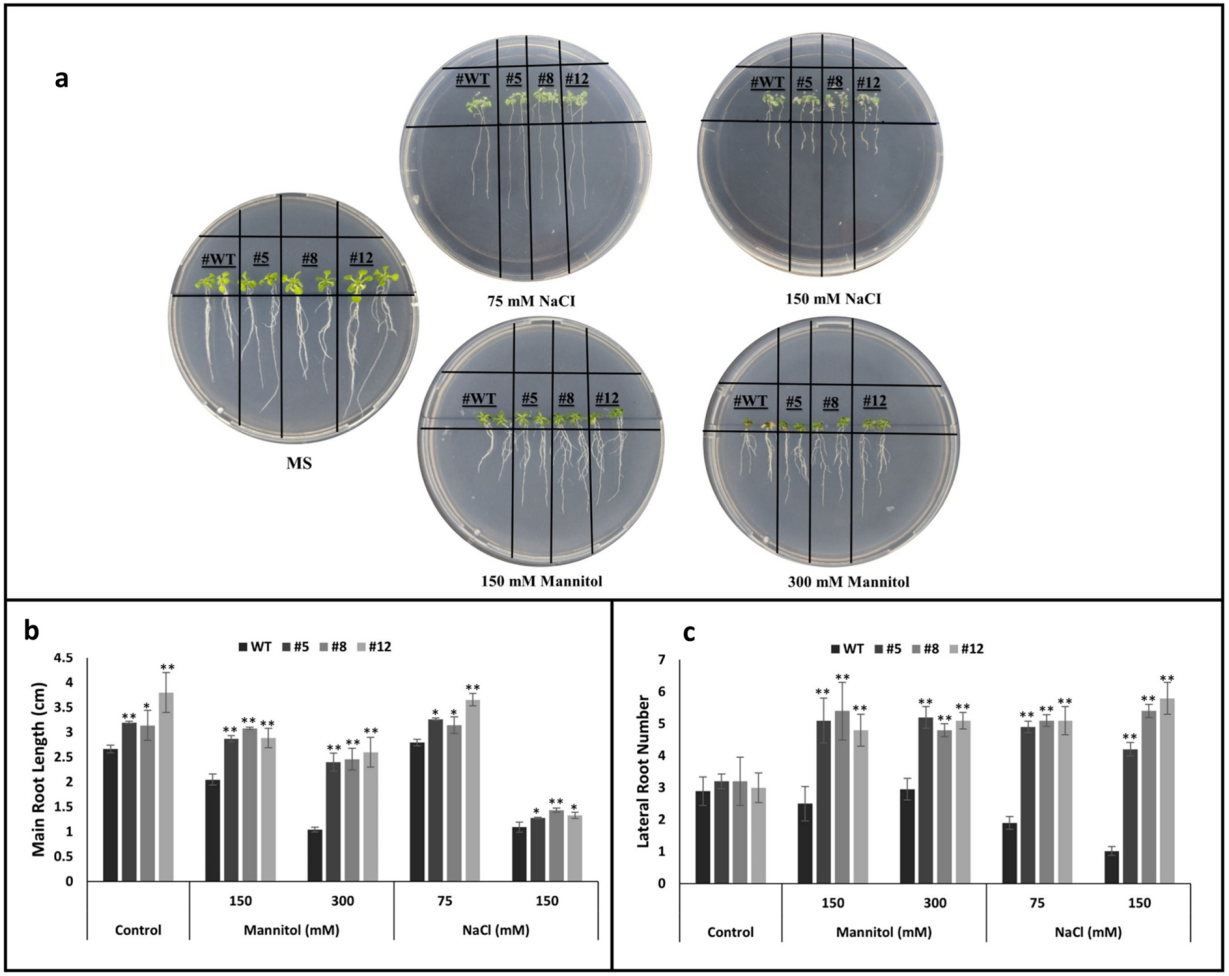
Effect of *PvMLP19* overexpression on root development under salinity and osmotic stress. Panel (**a**) provides a general representation of root development under control (1/2 MS), mannitol (150 mM and 300 mM), and NaCl (75 mM and 150 mM) conditions. Panel (**b**) shows main root lengths (cm) of WT and PvMLP19 transgenic lines (#5, #8, and #12) from the T3 generation under the same conditions. Panel (**c**) displays the number of lateral roots under the same conditions. Error bars indicate the mean ± standard deviation (SD) from three independent biological experiments. Statistical significance was evaluated using Student’s *t* test, with *p < 0.05 and **p < 0.01 denoting significant and highly significant differences, respectively (color figure online)

Root development of WT and *PvMLP19* transgenic lines (#5, #8, and #12) from the T3 generation was assessed under control conditions (1/2 MS) and stress induced by mannitol (150 mM and 300 mM) and NaCl (75 mM and 150 mM) (Fig. 4a). Under control conditions, the average primary root lengths were 2.66 cm for WT, 3.2 cm for line 5, 3.14 cm for line 8, and 3.8 cm for line 12. Under 150 mM mannitol, root lengths decreased to 2.05 cm, 2.87 cm, 3.08 cm, and 2.89 cm, respectively. At 300 mM mannitol, transgenic lines displayed significantly enhanced root development, with average root lengths of 1.04 cm for WT, 2.4 cm for line 5, 2.46 cm for line 8, and 2.6 cm for line 12 (Fig. 4b). NaCl stress similarly influenced root growth. At 75 mM NaCl, WT roots reached 2.8 cm, while lines 5, 8, and 12 measured 3.26 cm, 3.14 cm, and 3.7 cm, respectively. At 150 mM NaCl, the primary root lengths decreased to 1.1 cm in WT, 1.28 cm in line 5, 1.44 cm in line 8, and 1.33 cm in line 12 (Fig. 4b). Lateral root numbers were also analyzed (Fig. 4c). WT plants produced an average of 2.89, 2.5, 2.95, 1.9, and 1.02 lateral roots under control, 150 mM mannitol, 300 mM mannitol, 75 mM NaCl, and 150 mM NaCl conditions, respectively. In contrast, transgenic lines demonstrated significantly higher lateral root production. Line 5 exhibited averages of 3.2, 5.1, 5.2, 4.9, and 4.2, while line 8 produced 3.2, 5.4, 4.8, 5.1, and 5.4 lateral roots, and line 12 showed averages of 3.0, 4.8, 5.1, 5.1, and 5.8, respectively (Fig. 4c). These results suggest that *PvMLP19* overexpression enhances root development and lateral root formation under both mannitol and NaCl stress conditions. Overall, the overexpression of *PvMLP19* resulted in delayed germination, but significantly promoted root development under osmotic and salinity stress, indicating improved stress tolerance and adaptability compared to WT plants.

### Overexpression of *PvMLP19* enhances drought resilience

**Fig. 5:**
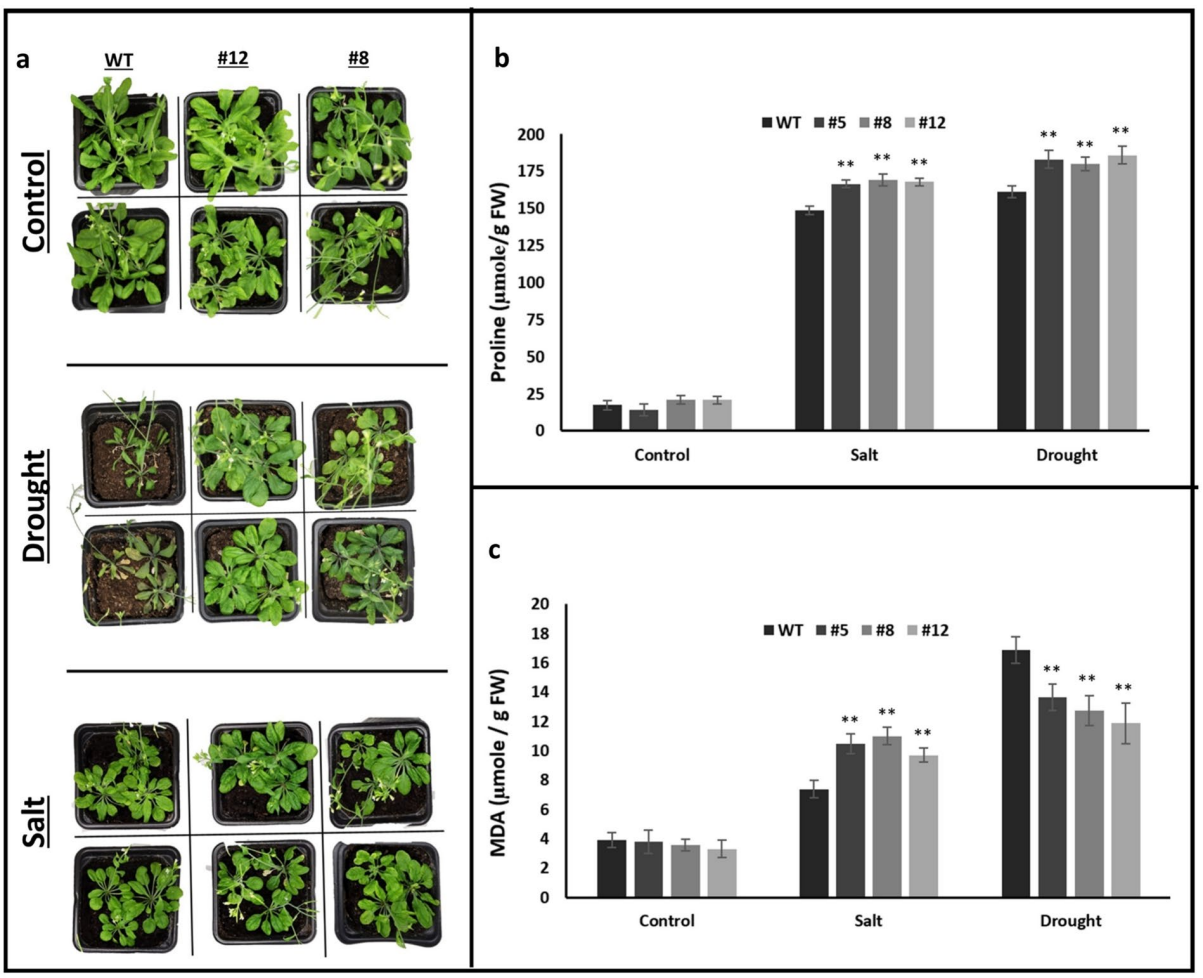
After 21 days of growth, seedlings were subjected to 10 days of drought stress (water deprivation) and 200 mM NaCl treatment. Panel (**a**) provides a morphological comparison of WT plants and *PvMLP19* transgenic lines (#5, #8, and #12) from the T3 generation. Panel (**b**) shows free proline content (µg g^−1^ FW) in WT and transgenic lines under control, salt, and drought conditions. Panel (**c**) displays MDA content (µmol g^−1^ FW) under the same conditions. Data represent the mean ± standard deviation (SD) from three independent biological replicates. Asterisks denote statistically significant differences between WT and transgenic lines (*p < 0.05, **p < 0.01) (color figure online)

To evaluate the salt and drought stress response, 21-day-old *Arabidopsis thaliana* plants grown under optimal irrigation were exposed to 10 days of water deprivation and 200 mM NaCl treatments. Figure 5 illustrates the pot experiment results, highlighting proline and MDA contents measured in WT and *PvMLP19* transgenic lines (#5, #8, and #12) from the T3 generation under control, salt, and drought conditions. No difference in proline content was observed under control conditions. Under 200 mM NaCl stress, WT plants accumulated an average of 148 µg g^−1^ FW proline, while transgenic lines showed higher accumulation: line 5 produced 166 µg g^−1^ FW, line 8 produced 169 µg g^−1^ FW, and line 12 accumulated 168 µg g^−1^ FW. Under 10 days of drought stress, WT plants accumulated 161 µg g^−1^ FW proline, while transgenic lines exhibited increased levels: line 5 at 183 µg g^−1^ FW, line 8 at 180 µg g^−1^ FW, and line 12 at 186 µg g^−1^ FW (Fig. 5B). No differences were observed among transgenic lines, but they exhibited significantly higher proline levels compared to WT. MDA content was measured spectrophotometrically, with no variation under control conditions. Under 200 mM NaCl stress, MDA levels in transgenic lines 5, 8, and 12 were 10.48, 11.01, and 9.71 µmol g^−1^ FW, respectively, significantly higher than the 7.37 µmol g^−1^ FW recorded in WT plants. Conversely, under 10 days of drought stress, WT plants exhibited higher MDA levels at 16.88 µmol g^−1^ FW compared to lines 5 (13.65 µmol g^−1^ FW), 8 (12.75 µmol g^−1^ FW), and 12 (11.88 µmol g^−1^ FW), indicating significantly lower lipid peroxidation and oxidative damage in transgenic lines (Fig. 5c). These results suggest that PvMLP19 overexpression enhances stress resilience by increasing proline accumulation and reducing oxidative damage under drought conditions, despite higher lipid peroxidation under salt stress.

### Gene expression analysis of stress-responsive markers in *PvMLP19* transgenic lines under salt and drought stress

**Fig. 6:**
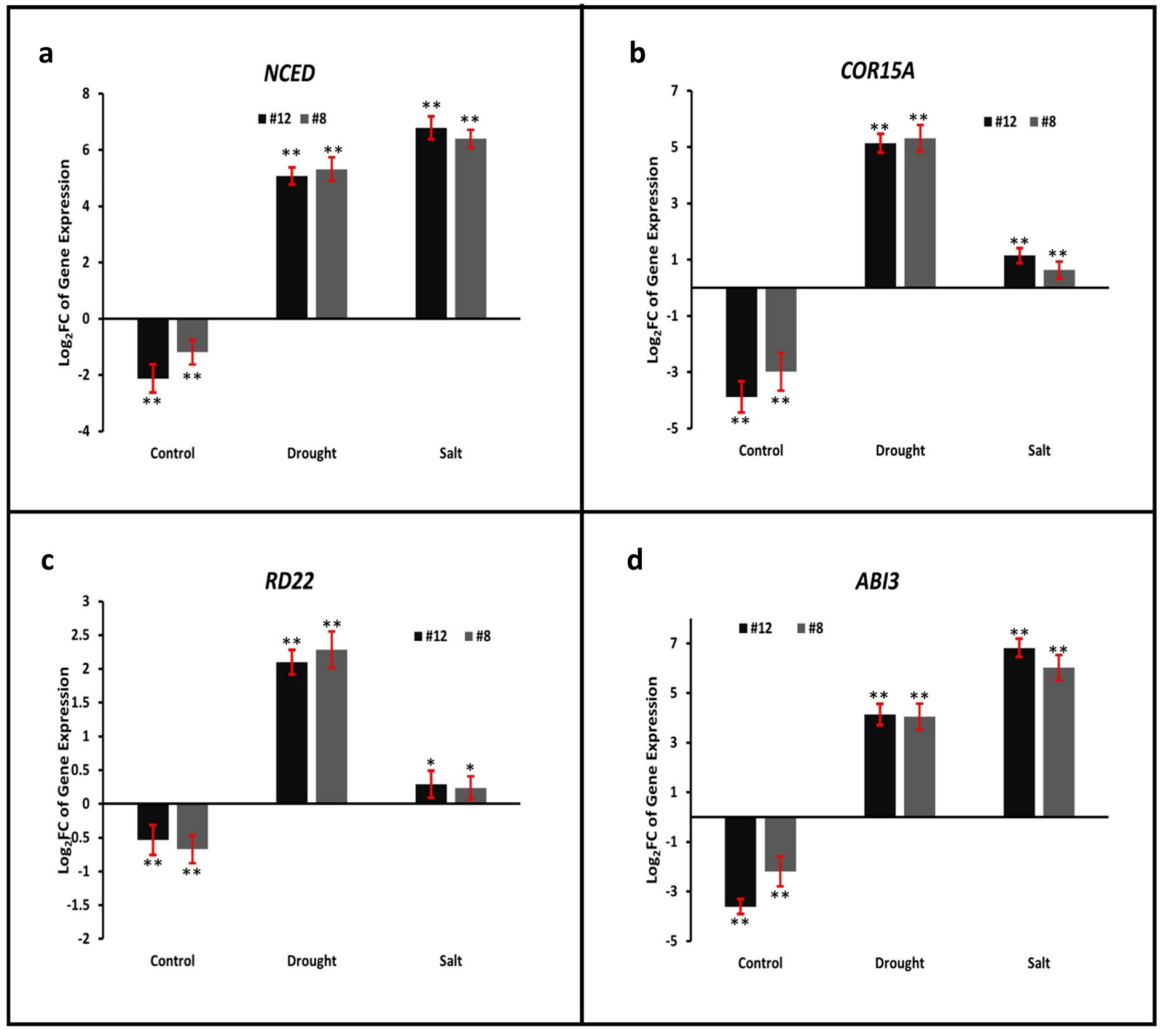
Expression patterns of stress and ABA-regulated genes influenced by *PvMLP19*. Relative expression levels of drought and salt-responsive genes were quantified in Arabidopsis WT and PvMLP19 transgenic lines (#8 and #12) from the T3 generation. Panel (**a**) shows the expression levels of the NCED gene, **b** COR15 A, **c** RD22, and **d** ABI3 genes. The reference gene EF-1α was used for normalization, and expression levels were calculated using the 2^−ΔΔ*Ct*^ method, with 2^−ΔΔ*Ct*^ values converted to Log_2_ FC compared to the WT. Error bars represent the mean ± standard deviation (SD) from three independent biological replicates. Asterisks denote statistically significant differences between WT and transgenic lines (*p < 0.05, **p < 0.01)

Gene expression analysis of key stress-responsive markers in Arabidopsis WT and *PvMLP19* transgenic lines (#8 and #12) from the T3 generation is illustrated in Fig. 6. The *NCED* gene, a crucial component in abscisic acid biosynthesis, exhibited pronounced upregulation in transgenic lines under drought and salt stress conditions. Specifically, Log_2_ FC values reached 5.32 and 6.40 in line 8 and 5.07 and 6.79 in line 12 for drought and salt stress, respectively (Fig. 6a). Similarly, the *COR15 A* gene showed increased expression in lines 8 and 12 compared to the WT, with Log_2_ FC values of 5.31 and 5.14 under drought and 0.64 and 1.15 under salt stress, respectively (Fig. 6b). The *RD22* gene exhibited a similar trend, with increased expression in transgenic lines under drought stress (Log_2_ FC: 2.29 and 2.1 for lines 8 and 12) and a modest increase under salt stress (Log2 FC: 0.24 and 0.29 for lines 8 and 12) compared to the WT (Fig. 6c). The *ABI3* gene showed the highest upregulation, with Log_2_ FC values of 4.05 and 4.14 under drought and 6.03 and 6.82 under salt stress for lines 8 and 12, respectively (Fig. 6d). These results suggest that *PvMLP19* overexpression enhances the expression of stress-responsive and ABA-regulated genes, potentially contributing to improved stress tolerance in transgenic lines under drought and salt stress conditions.

**Fig. 7:**
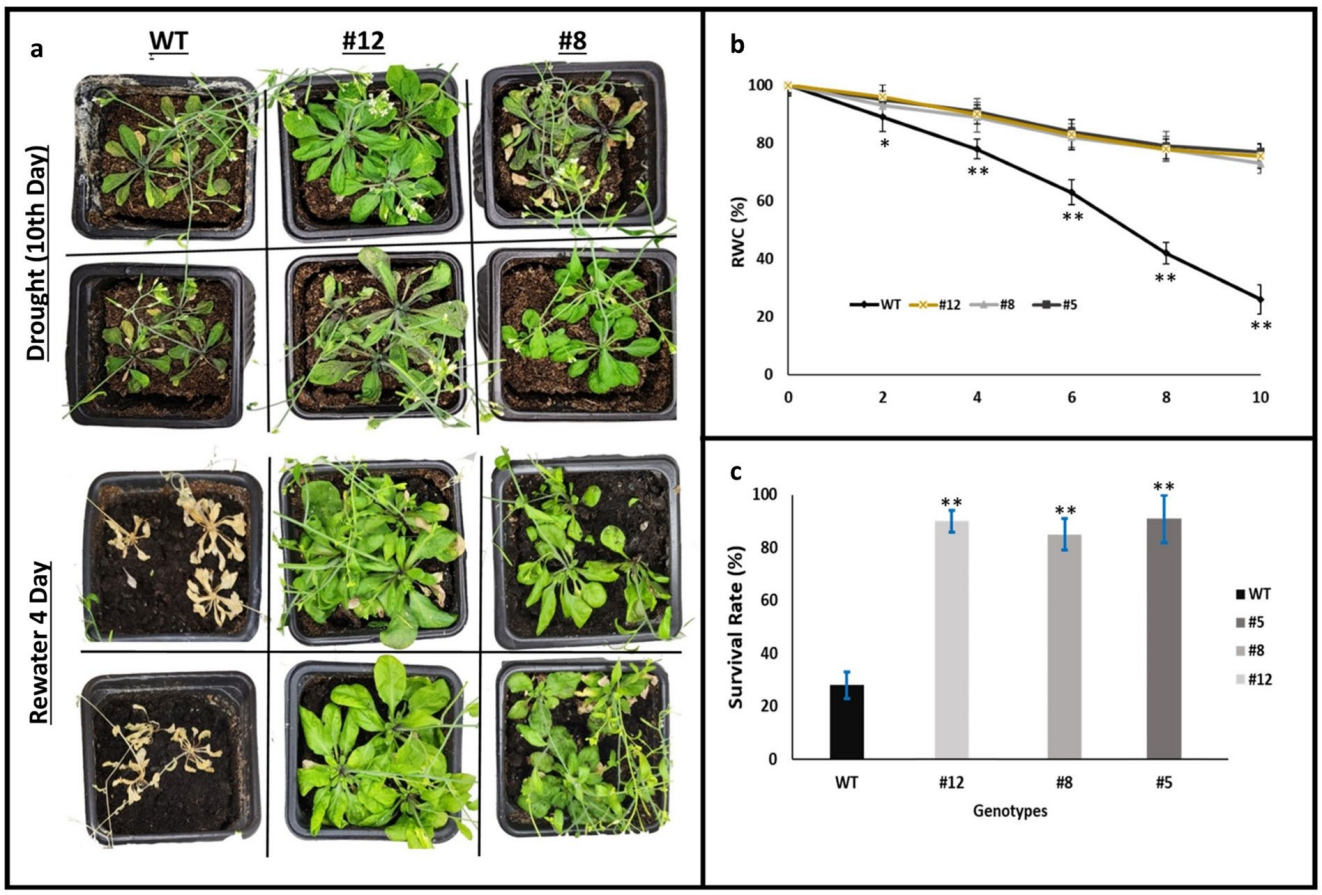
Recovery of PvMLP19 transgenic lines following drought stress. Panel (**a**) provides a morphological comparison of WT and PvMLP19 transgenic lines (#8 and #12) from the T3 generation after rewatering following drought stress, showing leaf yellowing and curling in WT plants and reduced damage in transgenic lines. Panel (**b**) shows the relative water content (%) of detached leaves during dehydration treatment for WT and *PvMLP19* transgenic lines (average of lines 8 and 12). Panel (**c**) displays survival rates (%) after rewatering for WT and transgenic lines (#5, #8, and #12). Data represent the mean ± standard deviation (SD) from three independent biological replicates. Asterisks denote statistically significant differences between WT and transgenic lines (*p < 0.05, **p < 0.01) (color figure online)

To evaluate recovery following drought stress, WT and *PvMLP19* transgenic lines (#5, #8, and #12) from the T3 generation were rewatered after a period of water scarcity. Observations indicated that water scarcity caused significant damage to the plants. As shown in Fig. 7a, WT plants exhibited severe stress symptoms, including leaf yellowing and curling, while *PvMLP19* transgenic lines (#8 and #12) showed significantly less damage. During dehydration treatment, detached leaves of WT plants lost water at a significantly faster rate compared to transgenic lines, with *PvMLP19* transgenic lines (#8 and #12) retaining a higher relative water content (83%) compared to WT plants (20%) (Fig. 7b). Survival rates were also significantly improved in transgenic lines, with line 5 showing an 89% survival rate, line 8 showing an 83% survival rate, and line 12 achieving 87%, in contrast to the 30% survival rate observed in WT plants (Fig. 7c). These results suggest that *PvMLP19* overexpression enhances drought stress recovery by improving water retention and survival rates in transgenic lines compared to WT plants. Overall, the overexpression of *PvMLP19* significantly enhances transgenic plant tolerance to drought by promoting proline accumulation, reducing oxidative stress, preserving higher relative water content, and improving survival rates under stress conditions while also resulting in the upregulation of key stress-responsive genes, further emphasizing the enhanced drought and salt stress tolerance in transgenic plants.

### Upregulation of auxin-responsive genes in *PvMLP19* overexpression lines under drought and salt stress conditions

**Fig. 8:**
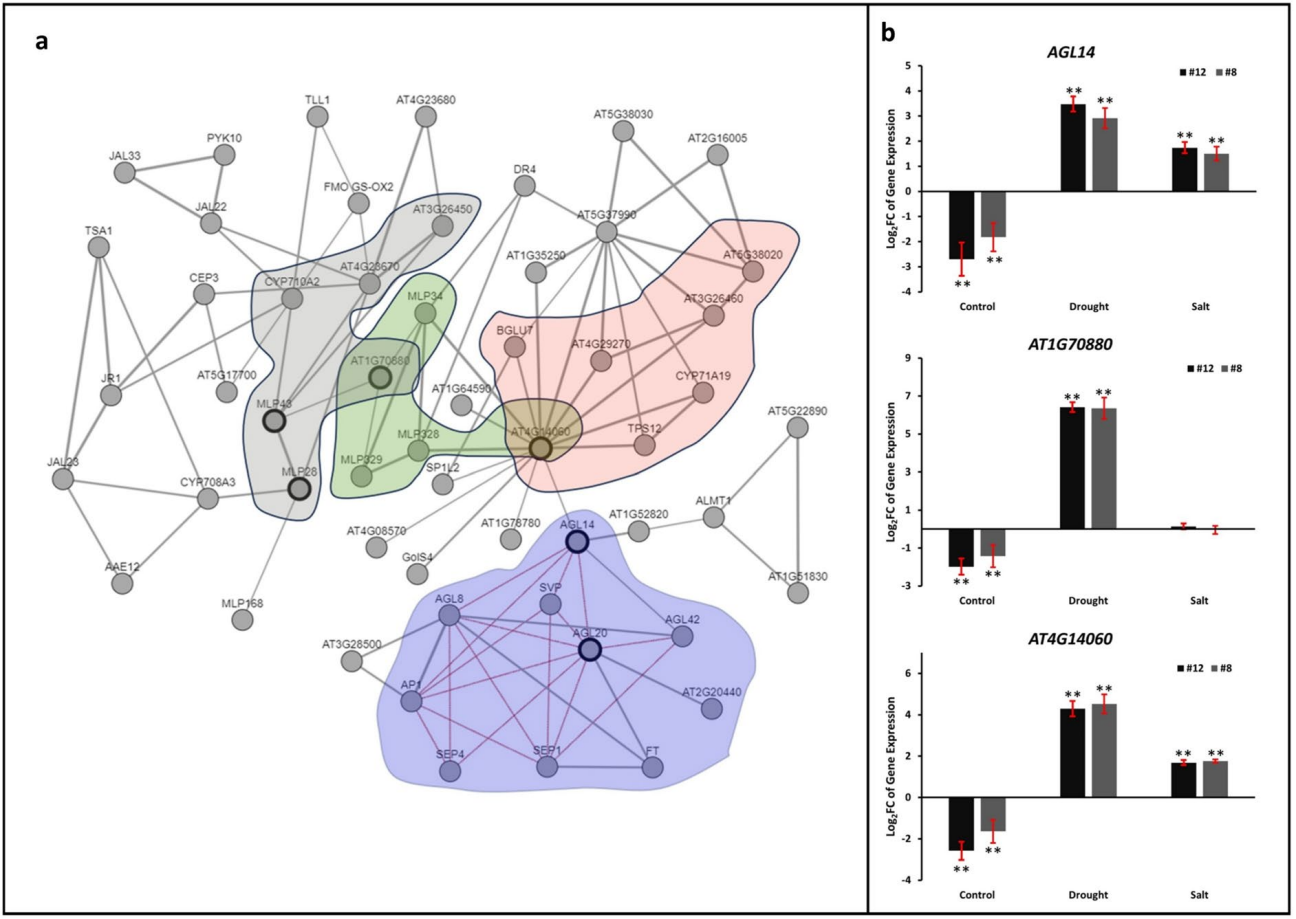
Upregulation of auxin-responsive genes in *PvMLP19* overexpression lines under drought and salt stress. **a** Gene regulatory network (GRN) of *AtMLP43*, the Arabidopsis ortholog of *PvMLP19*, constructed using the ATTED-II database (Obayashi et al. 2007). Red lines represent protein–protein interactions (PPIs), and black lines indicate co-expression relationships (Pearson correlation > 0.7). Four gene clusters are shown in gray (AGL14), purple (AT1G70880), pink (AT4G14060), and green. **b** Expression levels (Log2 FC) of AGL14, AT1G70880, and AT4G14060 in WT and PvMLP19 transgenic lines (#8 and #12) under control, drought, and salt (200 mM NaCl) conditions, relative to WT, validated by qRT-PCR. AGL14, a MADS-box transcription factor, regulates auxin transport via PIN genes (Melzer et al. 2008), while AT1G70880 and AT4G14060, polyketide cyclase/dehydrase proteins, are co-expressed with auxin-responsive genes (Obayashi et al. 2007). Data represent the mean ± standard deviation (SD) from three independent biological replicates. Asterisks denote significant differences (*p < 0.05, **p < 0.01) (color figure online)

To explore PvMLP19’s functional relationships, a co-expression network was constructed for its Arabidopsis ortholog AtMLP43 (AT1G70890) using the ATTED-II NetworkDrawer tool (Obayashi et al. 2007; v11.0) (Fig. 8a). Bidirectional BLASTP alignments identified PvMLP19 (Phvul.011G183900) as orthologous to AtMLP43 (BIT score: 104, e-value: 9e−29). The network integrated co-expression data (Pearson correlation > 0.7) and protein–protein interactions (PPIs) from the STRING database (score > 400). Three genes—AGL14 (AT4G11880), AT1G70880, and AT4G14060—were selected for qRT-PCR validation in PvMLP19-overexpressing lines due to their roles in auxin-related pathways. AGL14, a MADS-box transcription factor, regulates auxin transport via PIN gene modulation (Melzer et al. 2008), while AT1G70880 and AT4G14060, members of the polyketide cyclase/dehydrase family, are co-expressed with auxin-responsive genes in roots, suggesting indirect involvement in auxin signaling (Obayashi et al. 2007). qRT-PCR analysis in transgenic lines (#8 and #12) confirmed significant upregulation of these genes under drought and salt stress (Fig. 8b), validating their relevance to PvMLP19-mediated stress responses.

To explore PvMLP19’s functional relationships, a co-expression network was constructed for its Arabidopsis ortholog AtMLP43 (AT1G70890) using the ATTED-II NetworkDrawer tool (Obayashi et al. 2007) (Fig. 8a). Bidirectional BLASTP alignments identified PvMLP19 (Phvul.011G183900) as orthologous to AtMLP43 (BIT score: 104, e-value: 9e−29). The network integrated co-expression data (Pearson correlation > 0.7) and protein–protein interactions (PPIs) from the STRING database (score > 400). Three genes—AGL14 (AT4G11880), AT1G70880, and AT4G14060—were selected for qRT-PCR validation in PvMLP19-overexpressing lines due to their roles in auxin-related pathways (Fig. 8b). *AGL14* (AT4G11880) is a MADS-box transcription factor involved in the vegetative-to-generative transition and root development, interacting with auxin pathways; *AT1G70880* is a polyketide cyclase/dehydrase and lipid transport superfamily protein involved in response to biotic stimulus and defense response, expressed in roots; *AT4G14060* is a polyketide cyclase/dehydrase and lipid transport superfamily protein. The network illustrates the connection between *PvMLP19* and the expression of the genes *AGL14, AT1G70880,* and *AT4G14060*, with red lines between nodes representing protein–protein interactions and black lines indicating co-expression relationships. In transgenic line 12, the expression of the *AGL14* gene was − 2.7, 3.45, and 1.74 Log_2_ FC under control, drought, and salt stress conditions, respectively, compared to WT, while for line 8, the Log_2_ FC values were − 1.825, 2.92, and 1.51, respectively. The expression of *AT1G70880* in line 12 was − 1.99, 6.41, and 0.14 Log_2_ FC, while in line 8, it was − 1.43, 6.35, and −0.05 under the same conditions. For the *AT4G14060* gene, expression under control conditions was − 2.58 in line 12 and − 1.64 in line 8, but under drought stress, expression significantly increased to 4.29 and 4.52 Log_2_ FC, respectively, in these lines, with elevated expression persisting under salt stress at 1.69 and 1.77 Log_2_ FC for lines 12 and 8, respectively (Fig. 8b). Under control conditions, *AGL14, AT1G70880,* and *AT4G14060* were downregulated, whereas all three genes were strongly upregulated under drought stress. Under salt stress, *AGL14* and *AT4G14060* exhibited substantial upregulation, while *AT1G70880* showed minimal change. These results suggest that *PvMLP19* enhances the expression of stress-inducible auxin-responsive genes under stress conditions, contributing to improved stress tolerance in Arabidopsis by modulating crucial regulatory pathways.

## Discussion

The identification of 34 PR10 family proteins in the *Phaseolus vulgaris* v2.1 genome represents a significant step in uncovering this important legume species'molecular complexity and functional diversity (Feki et al. 2024). Through comprehensive phylogenetic analysis, these proteins were categorized into three major subfamilies: 3 in the PBP/NCS subfamily, 10 in the PR10/Bet v1-like subfamily, and 21 members in the MLP subfamily. MLP genes have been recognized as contributors to various aspects of plant growth, development, and responses to both biotic and abiotic stresses across numerous plant species (Chen and Dai 2010; Yang et al. 2015; Carella et al. 2016; He et al. 2020; Zhang et al. 2018). This study focused on the responses of PR10 family members in common bean, as previously characterized by Feki et al. (2022), under major abiotic stresses, specifically drought and salt. Prior research has indicated that MLPs may play roles in mitigating the adverse effects of these stresses, highlighting their potential in stress adaptation mechanisms.

RNA-seq analysis revealed significant variability in the expression patterns of *PvPR10* genes, with both upregulation and downregulation observed across different subfamilies in response to stress stimuli (Fig. 1). To validate these findings, qRT-PCR analysis was performed on selected *PvPR10* genes—*PvPR10-1, PvNCS, PvMLP19,* and *PvMLP21*—using RNA extracted from common bean roots subjected to various abiotic and phytohormone treatments (Fig. 2). The qRT-PCR results aligned closely with the RNA-seq data, suggesting that *PvPR10-1, PvNCS, PvMLP19,* and *PvMLP21* may contribute to plant resilience against drought and salt stress. These observations are consistent with studies in other species, such as *Oryza sativa*, where RSOsPR10, a rice PR10 protein, exhibited rapid induction in roots under similar stress conditions, including salt, drought, and fungal infections (Hashimoto et al. 2004). Such parallels suggest a conserved role for PR10 proteins across plant species in enhancing stress tolerance, though the specific mechanisms underlying PvMLP19’s function require further investigation.

Among the PR10 family members, *Phavu_011G183900 (PvMLP19)* emerged as a prominent candidate due to its notable induction under both salt and drought conditions in common bean (Figs. 1 and 2). Functional characterization of PvMLP19 in transgenic Arabidopsis revealed its multifaceted role in stress adaptation. Overexpression lines exhibited delayed germination under moderate to high mannitol and NaCl concentrations (Fig. 3), suggesting a protective mechanism during early development. In pot experiments, PvMLP19-overexpressing plants displayed enhanced drought tolerance, characterized by improved morphology, higher proline accumulation (Fig. 5b), reduced MDA levels (Fig. 5c), and higher relative water content and survival rates post-rewatering (Fig. 7). These traits indicate lower oxidative damage and improved water retention, underscoring PvMLP19’s potential in enhancing drought resilience (Kavas et al. 2013; Aksoy 2008). 

PvMLP19’s stress tolerance appears mediated through ABA and auxin signaling pathways. Upregulation of ABA-related genes under stress suggests a positive regulatory role in ABA-mediated responses (Fig. 6; Campalans et al. 1999; Cardoso et al. 2020), though additional physiological data (e.g., ABA quantification, stomatal responses) are needed to confirm this. Similarly, enhanced root growth and lateral root numbers under stress (Fig. 4) align with upregulated auxin-responsive genes (Fig. 8). Roots are typically the primary site of stress perception, responding to water deficits once a critical threshold is reached. Auxin, a key phytohormone, plays a central role in regulating plant responses to salinity and drought stresses, particularly through its influence on lateral root formation—an essential agronomic trait that shapes plant architecture and impacts crop productivity under environmental stresses (Forde 2002; De Smet et al. 2006). In this study, PvMLP19-overexpressing lines demonstrated enhanced root growth and increased lateral root numbers under moderate and high mannitol and NaCl stress conditions compared to WT plants (Fig. 4). Auxin biosynthesis primarily occurs in aboveground tissues, such as shoot apices and young leaves, via YUCCA synthesis genes, and is redistributed to roots through transport proteins, including auxin influx carriers (AUX1/LAX family) and efflux carriers (PIN proteins) (Blakeslee et al. 2005; Zažímalová et al. 2010; Péret et al. 2012). The polarity of PIN efflux carriers at the plasma membrane determines the directionality of auxin flux and its spatial distribution in plant tissues (Wisniewska et al. 2006). Gene regulatory network analysis linked PvMLP19 to auxin-responsive genes, including AGL14, AT1G70880, and AT4G14060 (Fig. 8). AGL14, a MADS-box transcription factor, modulates auxin transport by regulating PIN gene expression, critical for root development (Melzer et al. 2008; Forde 2002; De Smet et al. 2006). AT1G70880 and AT4G14060, co-expressed with auxin-responsive genes in roots, suggest indirect roles in auxin-mediated stress responses (Obayashi et al. 2007). Their upregulation in PvMLP19-overexpressing lines under stress supports PvMLP19’s contribution to root adaptation.

Comparative studies highlight shared and unique roles of MLP proteins. GhMLP28 from Gossypium hirsutum enhances osmotic stress tolerance (Chen and Dai 2010), while PsnMLP5 in *Populus simonii* × *P. nigra* responds to combined stresses via ABA signaling (Sun et al. 2024). AtMLP43 in Arabidopsis regulates ABA signaling for drought tolerance (Wang et al. 2016a, b). Unlike RSOsPR10 in rice, which responds to both biotic and abiotic stresses (Hashimoto et al. 2004), PvMLP19’s role in biotic stress remains unexplored, limiting our understanding of its full stress-response profile. This distinction underscores the functional diversity within the PR10 family and the need for further research to elucidate PvMLP19’s mechanisms.

Constitutive overexpression of *PvMLP19* using the 35S promoter in transgenic Arabidopsis unexpectedly led to reduced expression of ABA-regulated (*NCED3, RD22, COR15 A, ABI3*) and auxin-responsive genes (*AGL14, AT1G70880, AT4G14060*) under control conditions, despite the promoter’s continuous activity (Figs. 6 and 8). This downregulation may stem from disrupted hormone homeostasis, as ectopic *PvMLP19* expression could alter ABA or auxin levels, triggering feedback suppression of hormone-responsive genes, similar to how *GH3* overexpression in citrus reduced auxin levels and downregulated Aux/IAA genes (Zou et al. 2019). Alternatively, *PvMLP19* may act as a regulatory factor repressing ABA and auxin signaling, similar to *ZFP3* in Arabidopsis, which suppresses ABA-inducible genes like *RAB18* and *ABI4* when overexpressed (Joseph et al. 2014). Additionally, ABA- and auxin-responsive genes typically exhibit low basal expression in unstressed conditions, as seen with *RD22* and *COR15 A*, which remain silent without stress signals (Guo et al. 2014; Wang et al. 2016a, b). PvMLP19’s overexpression likely reinforces this repression, possibly through enhanced chromatin-level or protein-level suppressors, such as histone deacetylases (HD2 A/HD2B) that keep ABA-responsive genes off in non-stress conditions (Han et al. 2023). Similarly, Aux/IAA proteins repress auxin-responsive genes without auxin stimuli (Ulmasov et al. 1997). These mechanisms suggest *PvMLP19* creates a transcriptional “brake” on stress gene activation under control conditions, potentially via hormonal feedback or regulatory incompatibilities, while maintaining inducibility under stress (Saini et al. 2017; Xiong et al. 2001).

In conclusion, *PvMLP19* enhances drought and salt stress tolerance in Arabidopsis by promoting proline accumulation, reducing oxidative damage, and enhancing root development. Its context-dependent regulation of ABA and auxin pathways, coupled with its potential to optimize root architecture for improved crop resilience, underscores its promise. However, its unexplored biotic stress responses and mechanistic details require further investigation. These findings position *PvMLP19* as a candidate for genetic strategies to address climate-induced stresses.

## Conclusion

In summary, the findings of this study suggest that the PR10 protein family, particularly *PvMLP19*, may play a significant role in enhancing the tolerance of common beans (*Phaseolus vulgaris*) to drought and salinity stresses. Our results indicate that *PvMLP19* could function as a regulator of stress-related pathways, with its overexpression potentially leading to improved stress resilience, including enhanced proline accumulation, reduced oxidative stress, and higher water retention. Furthermore, *PvMLP19* overexpression was associated with promoted root development and delayed seed germination under stress conditions, which may contribute to plant survival in adverse environments. These findings position *PvMLP19* as a potential candidate for genetic improvement in crop species, particularly in the context of climate change, where drought and salinity stresses are becoming increasingly prevalent. However, limitations such as the lack of physiological data to confirm *PvMLP19’s* role in ABA-mediated pathways and the need for further validation of its interactions with transcription factors like AGL14 highlight areas for future research. By leveraging the regulatory potential of *PvMLP19*, future studies and breeding strategies could explore sustainable solutions to enhance crop productivity and food security. Overall, this study provides insights into the molecular mechanisms underlying stress tolerance in plants and opens avenues for further exploration of PR10 proteins in crop improvement programs.

## Supplementary Information

Below is the link to the electronic supplementary material.

Supplementary Figure Legend 1: Gene transfer and confirmation of transgenics A) Plasmid used for Agrobacterium transformation B) Confirmation of putative transgenics using hptII gene primers, with WT plants as a negative control C) Relative expression of *PvMLP19* gene in transgenic lines. Supplementary file1 (JPG 48 KB)

Supplementary file2 (XLSX 13 KB)

## Data Availability

All data generated or analyzed during this study are included in this published article (and its Supporting Information files). The materials used in our study are available under an MTA from the corresponding author upon reasonable request.
