# Supplementary figures and images for: Enhanced drought and salt stress tolerance in Arabidopsis via ectopic expression of the PvMLP19 gene
Source: Plant Cell Rep. 2025 May 22;44(6):130. doi: 10.1007/s00299-025-03520-y (PMC12098492; doi:10.1007/s00299-025-03520-y)

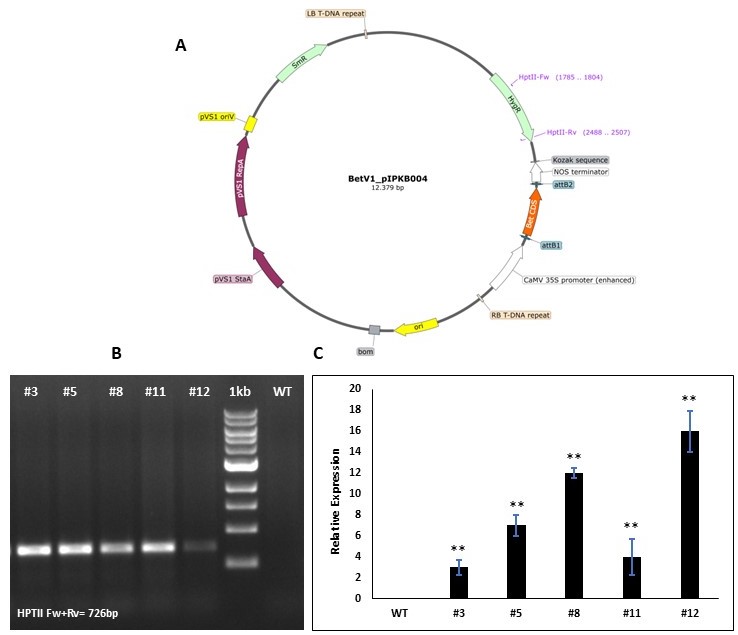

Supplement: Supplementary file 1 — Supplementary Figure Legend 1: Gene transfer and confirmation of transgenics A) Plasmid used for Agrobacterium transformation B) Confirmation of putative transgenics using hptII gene primers, with WT plants as a negative control C) Relative expression of PvMLP19 gene in transgenic lines. Supplementary file1 (JPG 48 KB) [file 299_2025_3520_MOESM1_ESM.jpg]
